# Pathological upgrading in prostate cancer treated with surgery in the United Kingdom: trends and risk factors from the British Association of Urological Surgeons Radical Prostatectomy Registry

**DOI:** 10.1186/s12894-019-0526-9

**Published:** 2019-10-17

**Authors:** Nicholas Bullock, Andrew Simpkin, Sarah Fowler, Murali Varma, Howard Kynaston, Krishna Narahari

**Affiliations:** 1 Division of Cancer and Genetics, Cardiff University School of Medicine, Cardiff, UK; 2 Department of Urology, Cardiff and Vale University Health Board, University Hospital of Wales, Cardiff, UK; 3 School of Mathematics, Statistics and Applied Mathematics, National University of Ireland, Galway, Ireland; 4 British Association of Urological Surgeons, London, UK; 5 Department of Cellular Pathology, Cardiff and Vale University Health Board, University Hospital of Wales, Cardiff, UK

**Keywords:** Gleason grade, Needle biopsy, Pathological, Prostate cancer, Radical prostatectomy

## Abstract

**Background:**

Accurate grading at the time of diagnosis if fundamental to risk stratification and treatment decision making in patients with prostate cancer. Whilst previous studies have demonstrated significant pathological upgrading and downgrading following radical prostatectomy (RP), these were based on historical cohorts and do not reflect contemporary patient selection and management practices. The aim of this national, multicentre observational study was to characterise contemporary rates and risk factors for pathological upgrading after RP in the United Kingdom (UK).

**Methods:**

All RP entries on the British Association of Urological Surgeons (BAUS) Radical Prostatectomy Registry database of prospectively entered cases undertaken between January 2011 and December 2016 were extracted. Those patients with full preoperative PSA, clinical stage, needle biopsy and subsequent RP pathological grade information were included. Upgrade was defined as any increase in Gleason grade from initial needle biopsy to pathological assessment of the entire surgical specimen. Statistical analysis and multivariate logistic regression were undertaken using R version 3.5 (R Foundation for Statistical Computing, Vienna, Austria).

**Results:**

A total of 17,598 patients met full inclusion criteria. Absolute concordance between initial biopsy and pathological grade was 58.9% (*n* = 10,364), whilst upgrade and downgrade rates were 25.5% (*n* = 4489) and 15.6% (*n* = 2745) respectively. Upgrade rate was highest in those with D’Amico low risk compared with intermediate and high-risk disease (55.7% versus 19.1 and 24.3% respectively, *P* < 0.001). Although rates varied between year of surgery and geographical regions, these differences were not significant after adjusting for other preoperative diagnostic variables using multivariate logistic regression.

**Conclusions:**

Pathological upgrading after RP in the UK is lower than expected when compared with other large contemporary series, despite operating on a generally higher risk patient cohort. As new diagnostic techniques that may reduce rates of pathological upgrading become more widely utilised, this study provides an important benchmark against which to measure future performance.

## Background

Despite first being described over fifty years ago, Gleason score has stood the test of time and remains one of the most powerful prognostic indicators in patients undergoing radical treatment with curative intent [1–5]. The Gleason grading system was updated in 2005 and again in 2014 following consensus conferences of the International Society of Urological Pathology (ISUP) [6, 7]. In the more recent update, the ISUP supported adoption of a validated Grade Group stratification system ranging from 1 (Gleason score ≤ 6) to 5 (Gleason score 9 or 10), to be used in conjunction with the overall Gleason system in order to simplify the number of grading categories and facilitate more accurate stratification of disease [6].

Despite advances in recent years, conventional diagnostic pathways employ transrectal ultrasound (TRUS) guided prostate biopsy to acquire systematic needle biopsies of the prostate, which has recently been shown to have a sensitivity of only 48% for the diagnosis of ‘clinically significant’ cancer, defined as Gleason score of at least 4 + 3 or a maximum core length of at least 6 mm [8]. Given the heterogeneity of disease it is not surprising that a significant proportion of cases are upgraded following radical prostatectomy (RP) compared with the initial TRUS biopsy [9–11]. This has wide ranging implications, as it may potentially lead to undertreatment of those that are undergraded by the initial biopsy, or conversely, overtreatment of those that have been overgraded. Furthermore, upgrading has been associated with adverse pathological outcomes, such as positive surgical margin status and biochemical recurrence [12, 13].

Whilst a small number of studies have reported pathological upgrading rates following RP in large patient populations, these utilised historical cohorts that underwent surgery prior to 2012 [10, 11]. Furthermore, neither involved participants from the United Kingdom (UK), thereby limiting the generalisability of findings to this patient population and the National Health Service. This is of particular importance as, owing to differences in screening practices across Europe and North America, a relatively high proportion of patients present with advanced disease at the time of diagnoses in the UK [14–18].

The objective of this large observational study was to characterise contemporary rates of pathological upgrading after RP in the UK and identify risk factors for upgrading within this population.

## Methods

### Case selection

Data for patients undergoing radical prostatectomy in the UK were uploaded prospectively by individual surgeons or institutions onto the British Association of Urological Surgeons (BAUS) Radical Prostatectomy Registry database. All patients that underwent RP between 1st January 2011 and 31st December 2016 were eligible for inclusion. Patients with missing data for key variables such as preoperative Prostate Specific Antigen (PSA), initial biopsy Gleason grade, clinical T stage or final RP Gleason grade were excluded, as were those that underwent salvage surgery.

### Pathological assessment

The time from initial biopsy to RP was not recorded in the database. However, in the UK the National Health Service Cancer Reform Strategy states that patients diagnosed with cancer should receive definitive treatment within 31 days of the decision to do so. This means that most patients will have undergone surgery within 1–2 months of the initial biopsy, thereby ameliorating potential for genuine grade progression between histological assessment of the biopsy and surgical specimen.

Whilst the exact type of initial prostate biopsy was not recorded, TRUS guided biopsy utilising an extended sampling approach was standard practice across the UK during the study period. All biopsies were evaluated and reported by a Consultant Pathologist with or without expertise in urological pathology. Almost all RP specimens were examined and reported by a Consultant Pathologist with expertise in urological pathology. No cases were re-reviewed for the purposes of this study. The primary outcome was upgrading, defined as any increase in Gleason grade between the initial biopsy and final RP histology.

### Statistical analysis

Continuous variables were reported using mean, median and range, and categorical variables as percentages. For unadjusted comparison between upgraded and non-upgraded cases, Chi-squared and t-tests were used to assess for statistically significant differences in categorical and continuous variables respectively. Logistic regression was used to formally determine the association of key preoperative variables and upgrading, with the odds ratio for each being reported alongside a 95% confidence interval and *p*-value. D’Amico risk category was not included in the mutually adjusted model, since it is a composite variable derived from PSA, clinical T-stage and biopsy Gleason grade (all of which were included in the model). All analyses were performed in R v3.5 (R Core Team (2018). R: A language and environment for statistical computing. R Foundation for Statistical Computing, Vienna, Austria).

## Results

### Patient demographics

17,598 of 30,424 patients that were entered onto the registry between 1st January 2011 and 31st December 2016 met full inclusion criteria. Table 1 demonstrates the patient and operative characteristics. Mean age and preoperative PSA were 63.2 years and 10.06 ng/ml respectively. The majority of cases were performed for D’Amico high risk disease (52.7%; *n* = 1766), with only 10% (*n* = 1766) for low risk disease. The proportion of cases performed each year increased from 2011 to 2016, owing to both a yearly increase in the number of cases entered onto the registry together with a reduction in the number of cases excluded on account of missing data each year.

**Table 1:** Patient, disease and operative characteristics

Patient and disease characteristics	
Age (years)	
Mean	63.22
Median	64
Range	35–92
Preoperative PSA (ng/ml)	
Mean	10.06
Median	7.85
Range	0–181
Preoperative biopsy ISUP Grade Group (%)	
1	3914 (22.2)
2	8328 (47.3)
3	2893 (16.4)
4	1427 (8.1)
5	1036 (5.9)
Preoperative clinical stage (%)	
T1	5435 (30.9)
T2	8311 (47.2)
T3	3839 (21.8)
T4	13 (0.10
Preoperative D’Amico risk group (%)	
Low risk	1766 (10.0)
Intermediate risk	6563 (37.3)
High risk	9269 (52.7)
Operative characteristics	
Year of surgery (%)	
2011	1001 (5.7)
2012	1159 (6.6)
2013	2259 (12.8)
2014	3783 (21.5)
2015	4566 (25.9)
2016	4830 (27.4)
Region (%)	
A	79 (0.4)
B	701 (4.0)
C	1653 (9.4)
D	2081 (11.8)
E	706 (4.0)
F	2084 (11.8)
G	1315 (7.5)
H	29 (0.2)
I	67 (0.4)
J	2093 (11.9)
K	1885 (10.7)
L	1736 (9.9)
M	745 (4.2)
N	1798 (10.2)
O	324 (1.8)
P	302 (1.7)

### Pathological upgrade rates

Absolute concordance between initial biopsy and pathological Gleason grade was 58.9% (*n* = 10,364), whilst upgrade and downgrade rates were 25.5% (*n* = 4489) and 15.6% (*n* = 2745) respectively. Table 2 demonstrates concordance between the initial biopsy and final RP histology as stratified by ISUP Grade Groups. Concordance was lowest in those patients with Grade Group 1 and 4 on initial biopsy (39.6 and 25.4% respectively), whilst highest in those patients with Grade Group 2 (76.6%). An increase by one ISUP Grade Group (for example, from Grade Group 1 on initial biopsy to Grade Group 2 on final RP histopathology) constituted the majority of upgrading events, with 319 (8.2%), 286 (3.4%) and 181 (6.3%) cases being upgraded by two or more Grade Groups in patients with Grade Group 1, 2 and 3 on initial biopsy respectively.

**Table 2:** Concordance between the initial biopsy and final RP histology when stratified by ISUP Grade Groups

RP ISUP Grade Group	Biopsy ISUP Grade Group									
	1 (≤6)		2 (3 + 4)		3 (4 + 3)		4 (8)		5 (9–10)	
	No.	%	No.	%	No.	%	No.	%	No.	%
1 (≤6)	1550	39.6	430	5.2	38	1.3	21	1.5	2	0.2
2 (3 + 4)	2045	52.2	6381	76.6	955	33.0	351	24.6	103	9.9
3 (4 + 3)	235	6.0	1231	14.8	1579	54.6	486	34.1	227	21.9
4 (8)	52	1.3	165	2.0	140	4.8	363	25.4	73	7.0
5 (9–10)	32	0.8	121	1.5	181	6.3	206	14.4	631	60.9
Total	3914	100	8328	100	2893	100	1427	100	1036	100

### Risk factors for pathological upgrading

**Fig. 1:**
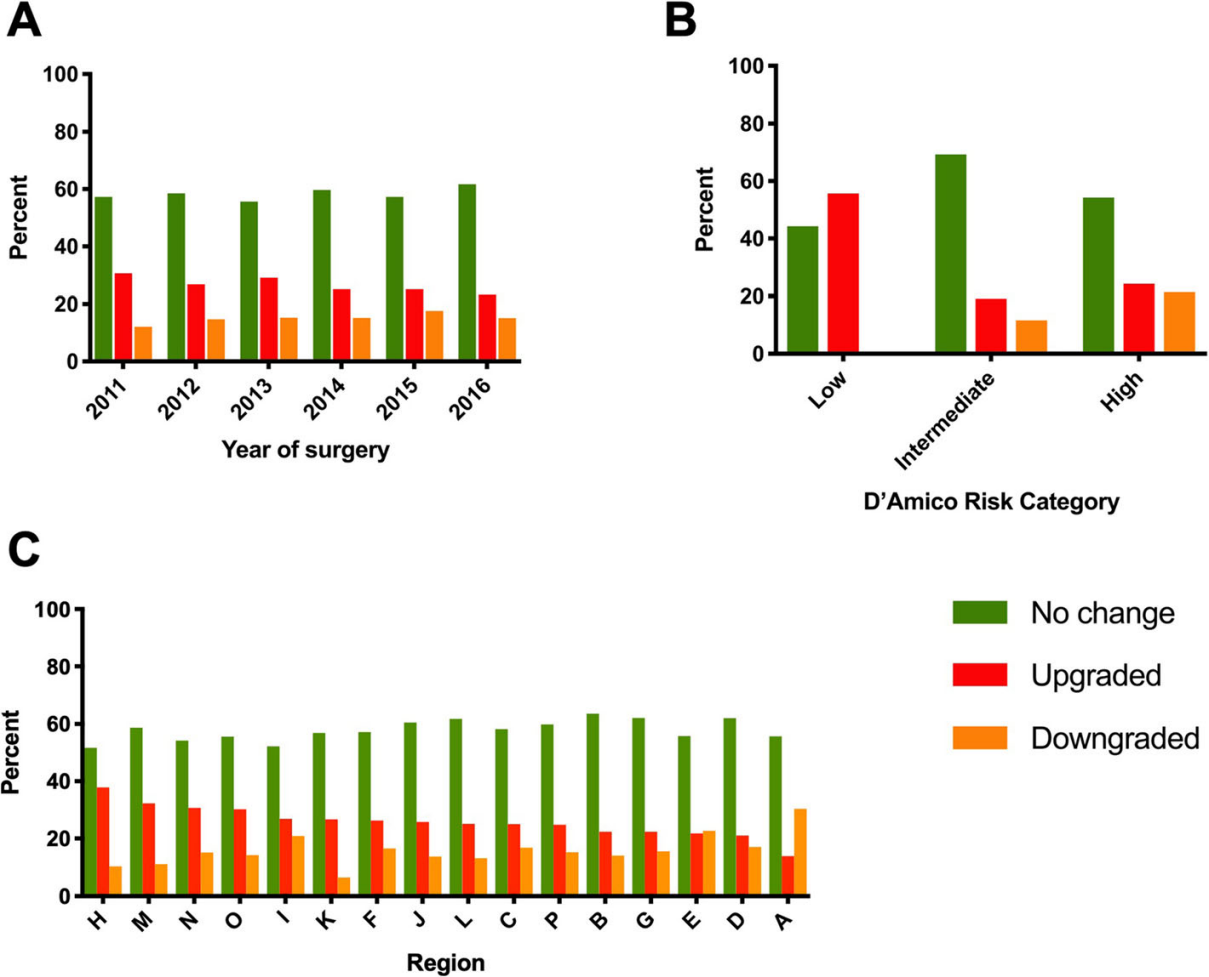
Grade change according to: **a**, year of surgery. **b**, D’Amico risk category. **c**, anonymised region, ranked in order of upgrade rate

Table 3 provides the association between patient and operative characteristics and pathological upgrading. Upgrading was associated with a higher preoperative PSA (10.8 ng/ml versus 9.81 ng/ml; *p* < 0.001) and reduced over time, with 30.6% of cases being upgraded in 2011 compared with 23.2% in 2016 (Fig. 1a; p < 0.001). Of note, upgrade rate was highest in those with D’Amico low risk compared with intermediate and high-risk disease (Fig. 1b; 55.7% versus 19.1 and 24.3% respectively, *P* < 0.001). There was considerable variation in upgrading between regions, with rates ranging from 13.9% in region A to 37.9% in region H (Fig. 1c; P < 0.001).

**Table 3:** Association between patient, disease and operative characteristics and pathological upgrading after RP

Characteristic	No upgrade (*n* = 13,109)	Upgrade (*n* = 4489)	*p*-value
Age (years)	63.2 (6.56)	63.3 (6.44)	0.159
Preoperative PSA (ng/ml)	9.81 (7.93)	10.8 (8.94)	< 0.001
Preoperative biopsy ISUP Grade Group			< 0.001
1	1550 (39.6%)	2364 (60.4%)	
2	6811 (81.8%)	1517 (18.2%)	
3	2572 (88.9%)	321 (11.1%)	
4	1213 (85.0%)	214 (15.0%)	
5	963 (93.0%)	73 (7.05%)	
Preoperative clinical stage			< 0.001
T1	3957 (72.8%)	1478 (27.2%)	
T2	6350 (76.4%)	1961 (23.6%)	
T3	2792 (72.7%)	1047 (27.3%)	
T4	10 (76.9%)	3 (23.1%)	
Preoperative D’Amico risk group			< 0.001
Low risk	782 (44.3%)	984 (55.7%)	
Intermediate risk	5307 (80.9%)	1256 (19.1%)	
High risk	7020 (75.7%)	2249 (24.3%)	
Year of surgery			< 0.001
2011	695 (69.4%)	306 (30.6%)	
2012	848 (73.2%)	311 (26.8%)	
2013	1602 (70.9%)	657 (29.1%)	
2014	2835 (74.9%)	948 (25.1%)	
2015	3420 (74.9%)	1146 (25.1%)	
2016	3709 (76.8%)	1121 (23.2%)	
Region			< 0.001
A	68 (86.1%)	11 (13.9%)	
B	544 (77.6%)	157 (22.4%)	
C	1239 (75.0%)	414 (25.0%)	
D	1643 (79.0%)	438 (21.0%)	
E	552 (78.2%)	154 (21.8%)	
F	1536 (73.7%)	548 (26.3%)	
G	1020 (77.6%)	295 (22.4%)	
H	18 (62.1%)	11 (37.9%)	
I	49 (73.1%)	18 (26.9%)	
J	1553 (74.2%)	540 (25.8%)	
K	1382 (73.3%)	503 (26.7%)	
L	1301 (74.9%)	435 (25.1%)	
M	505 (67.8%)	240 (32.2%)	
N	1246 (69.3%)	552 (30.7%)	
O	226 (69.8%)	98 (30.2%)	
P	227 (75.2%)	75 (24.8%)

Table 4 gives the results of the mutually adjusted logistic regression analysis for upgrading. After adjusting for differences in other characteristics, both increased age and preoperative PSA were associated with increased odds of pathological upgrading (OR 1.022; *p* < 0.001 per year and OR 1.026; p < 0.001 per ng/ml respectively). Higher initial biopsy ISUP Grade Groups were associated with lower odds of upgrading, whilst clinically T2 and T3 tumours had the highest odds of upgrading. Those cases performed in more recent years were associated with reduced odds of upgrading when compared with 2011, although all failed to reach significance. Likewise, despite apparent regional differences, almost all failed to reach significance.

**Table 4:** Mutually adjusted logistic regression analysis for pathological upgrading after RP

Characteristic	Odds ratio (95% confidence interval)	*p*-value
Age	1.022 (1.016–1.028)	< 0.001
Preoperative PSA	1.026 (1.022–1.031)	< 0.001
Preoperative biopsy ISUP Grade Group		
*1 (reference)*	1	
2	0.123 (0.112–0.135)	< 0.001
3	0.057 (0.049–0.065)	< 0.001
4	0.085 (0.072–0.101)	< 0.001
5	0.030 (0.023–0.039)	< 0.001
Preoperative clinical stage		
T1 *(reference)*	1	
T2	1.231 (1.120–1.353)	< 0.001
T3	1.990 (1.774–2.232)	< 0.001
T4	1.199 (0.275–5.237)	0.809
Year of surgery		
2011 *(reference)*	1	
2012	0.930 (0.749–1.156)	0.514
2013	1.084 (0.896–1.311)	0.408
2014	0.851 (0.710–1.021)	0.082
2015	0.935 (0.782–1.118)	0.462
2016	0.908 (0.759–1.086)	0.289
Region		
A	0.792 (0.403–1.556)	0.498
B	1.077 (0.857–1.353)	0.526
C	1.071 (0.906–1.265)	0.421
D	0.980 (0.834–1.152)	0.808
E	0.886 (0.704–1.115)	0.303
F	1.152 (0.984–1.348)	0.078
G	0.891 (0.743–1.068)	0.210
H	1.980 (0.840–4.669)	0.118
I	1.299 (0.688–2.452)	0.420
J *(reference)*^*a*^	1	
K	0.830 (0.706–0.976)	0.024
L	0.863 (0.731–1.020)	0.084
M	0.837 (0.678–1.033)	0.098
N	1.132 (0.963–1.330)	0.133
O	0.995 (0.741–1.338)	0.976
P	0.654 (0.477–0.897)	0.008

^a^Region J chosen as a reference owing to largest number of patients

## Discussion

This is the largest study to date exploring pathological upgrading after radical prostatectomy, as well as the first of its kind conducted within a large contemporary UK patient cohort. There are several potential reasons for differences occurring between initial biopsy Gleason grade and that of the RP specimen. Whilst genuine grade progression in the time between initial biopsy and RP is possible, this is unlikely and thus the majority of discrepancy occurs as a result of either sampling error or variation in pathological reporting.

Sampling error occurs when an area of high-grade tumour is missed by the initial needle biopsy, leading to undergrading. Conversely, the biopsy may oversample the high-grade component of a tumour. This scenario is well described, with many studies demonstrating conventional TRUS guided prostate biopsy to be poor at localising the index tumour and/or estimating true tumour grade [9–11, 19].

Variation in pathological reporting may result from the inherent subjectivity of tumour grading, microscopic interpretation issues and differences in rules used to report the Gleason score. Tumour grade is a morphological and biological continuum with arbitrary cut-offs, so a degree of variation is inevitable in borderline cases. Microscopic interpretation such as distinction between poorly formed glands of pattern 4 and tangential sectioning of pattern 3 glands is also subjective [10].

A number of authors have therefore sought to quantify the level of inter-observer agreement among pathologists [20, 21], with one recent study demonstrating kappa values of 0.61 for needle biopsies and 0.37 for RP specimens between the original pathology report and a ‘gold standard’ report issued by expert urological pathologists [22]. This study also recognised that the accuracy of Gleason grading may depend on the level of experience and training of the reporting pathologist [22]. Whilst the differing service structure means the findings of many of these US based studies cannot be completely applied to the UK, inter-observer variability will no doubt be partially responsible for some of the grade changes seen in the current series. For example, whilst several cases were upgraded following RP, a significant number were also downgraded, most notably from ISUP Grade Group 3 to 2 and from Grade Group 4 to either 3 or 2. In the UK many initial needle biopsies are reported in smaller District General Hospitals by pathologists who may or may not have expertise in urological pathology, whilst almost all RP specimens are reported in larger tertiary referral centres by expert urological pathologists. It is therefore possible that some of the observed overcalling of Gleason pattern 4 may have arisen due to lack of awareness among general UK pathologists of the 2014 ISUP consensus conference recommendation that occasional/seemingly poorly formed or fused glands are insufficient for a diagnosis of pattern 4 [6]. Finally, even when pathologists agree on the grade, they may report the Gleason score differently. For example, when cores show different Gleason scores, contemporary practice varies with either the overall (global) or worst Gleason score recorded for each biopsy series [7, 23, 24].

In this cohort of 17,598 patients we found overall upgrade and downgrade rates of 25.5 and 15.6% respectively. Interestingly upgrade rate was highest in those patients undergoing RP for low risk prostate cancer, as classified using D’Amico criteria that are widely used in UK practice [25]. Whilst this is somewhat expected given that this group comprises patients with Gleason 3 + 3 = 6 disease on initial biopsy, for whom the only change in grade can be upgrading, the rate of 55.7% is higher than reported in other comparable series. Whilst this may have implications for counselling men with low risk disease that are considering surveillance, we must be wary not to extrapolate this figure to all patients. In the UK, recent years have seen a reduction in the number of men with low risk disease that undergo radical treatment [14], which is reflected within our cohort (*n* = 1766; 10%). This means those patients with low risk disease that undergo RP are likely to have other ‘high risk’ features, such as large volume tumour.

This study also demonstrated a reduction in upgrading in more recent years, with the rate falling from 30.6% in 2011 to 23.2% in 2016. Furthermore, we also identified variation in upgrading between geographical regions, with rates ranging from 13.9% in region A to 37.9% in region H. However, when adjusting for differences in other preoperative variables, almost all differences between regions and years of surgery failed to reach significance. Collectively this means that such differences can be explained by variation in other factors such as increasing age, preoperative PSA and clinical stage, as well as decreasing biopsy ISUP Grade Group, all of which were found to be significant risk factors for upgrading in our mutually adjusted logistic regression model.

Whilst TRUS guided biopsy utilising an extended sampling approach was standard practice across the UK during the study period, the exact biopsy technique utilised in each case was not recorded in the registry. Recent years have seen a trend towards increased uptake of pre-biopsy mpMRI and contemporary diagnostic techniques such as mpMRI influenced biopsy strategy, template guided transperineal biopsy and mpMRI-TRUS fusion biopsy, which have been shown to reduce pathological upgrading after surgery [26–28]. Furthermore, these techniques have also been shown to reduce diagnosis of ‘clinically insignificant’ cancers, such as those that are Gleason 3 + 3 [29]. Collectively this may explain the trend towards a reduction in upgrading in seen in more recent years in this series. Furthermore, as these techniques continue to become more widely utilised, future rates of pathological upgrading are likely to decrease, thereby rendering this study an important benchmark against which to measure performance.

A number of other studies have explored upgrading and downgrading following RP. Whilst many of these utilised small historical cohorts, the results of the two largest and most recent series may be compared to the findings presented here. The first of these, published by Epstein et al. in 2012, reported grade change in 7643 US patients that underwent surgery between 2002 and 2010 [10]. Although similar in terms of age, the cohort had a lower PSA and higher proportion of T1 and Gleason 6 disease, thus representing a lower risk population. Interestingly the authors report an upgrade rate of 36.3% from Gleason 5–6 to a higher grade after RP, representing a higher concordance in this group compared with the present study (63.7% versus 39.6% respectively). The reasons for these differences are likely multifactorial but may be due to the small proportion of patients with initial biopsy Gleason 6 undergoing surgery within our cohort, along with the likelihood of this group possessing additional high-risk features that would have favoured this treatment approach. However, despite these differences, there was agreement that both increasing age and preoperative PSA were predictors of upgrading [10].

More recently, Danneman et al. reported upgrading within a cohort of 15,598 patients from the Swedish National Prostate Cancer Register that underwent RP between 2000 and 2012 [11]. Inclusion criteria restricted analysis to patients less than 70 with T1–2 disease and a serum PSA of < 20 ng/ml, thereby again comprising a lower risk population than represented here. The authors report an upgrade rate of 35% from Gleason 2–6 to a higher grade after RP, giving a concordance of 65% in this group. Once again this is much higher than the present study and may similarly be due to differences in patient demographics.

The strengths of this study lie in the large cohort and its origin from a national level data registry. Furthermore, unlike similar studies, all patients were treated between 2011 and 2016, thereby representing more contemporary patient selection and management practices. This is particularly relevant to the UK, where reducing numbers of patients with low risk disease are undergoing radical intervention each year [14].

This study does however have a number of limitations, including the surgeon/institution reported nature of the data registry. Although a large proportion of the total number of patients that undergo RP in UK are entered, some regions report few numbers and it is possible there are differences between those patients that are entered and those that are not. Another limitation is the amount of missing preoperative data, which led to the exclusion of a number of cases from the full analysis. This is a recognised issue with the BAUS Radical Prostatectomy Registry that must be acknowledged but has not prevented meaningful observations being reported [30, 31]. The type of initial biopsy performed was also unclear, as was whether the overall or worst Gleason score had been recorded in each case. However, these scores are different in only a minority of cases and previous studies have demonstrated that both are clinically comparable [32]. Finally, the registry lacked additional pathological parameters that have previously been linked to risk of upgrading, including prostate size [10, 12] and extent of cancer in the biopsy [10, 13].

## Conclusions

Pathological upgrading after RP remains an important consideration in the management of patients with prostate cancer. This large study demonstrates that overall upgrading in the UK is lower than expected, with risk factors including increasing age, preoperative PSA, and clinical stage. As new diagnostic techniques that may reduce rates of pathological upgrading become more widely utilised, this study will provide an important benchmark against which to measure performance.

## Data Availability

The BAUS Radical Prostatectomy Registry data that support the findings of this study are held centrally by the British Association of Urological Surgeons and are not publicly available. A request for use of data may be made via formal application to the BAUS Data & Audit Manager.

## Abbreviations

BAUS: British Association of Urological Surgeons
ISUP: International Society of Urological Pathology
mpMRI: multi-parametric magnetic resonance imaging
PSA: Prostate Specific Antigen
RP: Radical prostatectomy
TRUS: Transrectal ultrasound
UK: United Kingdom
US: United States of America
